# An automated toolbox for microcalcification cluster modeling for mammographic imaging

**DOI:** 10.1002/mp.17521

**Published:** 2024-11-21

**Authors:** Astrid Van Camp, Eva Punter, Katrien Houbrechts, Lesley Cockmartin, Renate Prevos, Nicholas W. Marshall, Henry C. Woodruff, Philippe Lambin, Hilde Bosmans

**Affiliations:** ^1^ Department of Precision Medicine GROW – Research Institute for Oncology and Reproduction Maastricht University Maastricht The Netherlands; ^2^ Department of Imaging and Pathology Division of Medical Physics & Quality Assessment KU Leuven Leuven Belgium; ^3^ Department of Radiology UZ Leuven Leuven Belgium; ^4^ Department of Radiology and Nuclear Medicine GROW – Research Institute for Oncology and Reproduction Maastricht University Medical Centre+ Maastricht The Netherlands

**Keywords:** mammography, microcalcification cluster, simulation

## Abstract

**Background:**

Mammographic imaging is essential for breast cancer detection and diagnosis. In addition to masses, calcifications are of concern and the early detection of breast cancer also heavily relies on the correct interpretation of suspicious microcalcification clusters. Even with advances in imaging and the introduction of novel techniques such as digital breast tomosynthesis and contrast‐enhanced mammography, a correct interpretation can still be challenging given the subtle nature and large variety of calcifications.

**Purpose:**

Computer simulated lesion models can serve to develop, optimize, or improve imaging techniques. In addition to their use in comparative (virtual clinical trial) detection experiments, these models have potential application in training deep learning models and in the understanding and interpretation of breast lesions. Existing simulation methods, however, often lack the capacity to model the diversity occurring in breast lesions or to generate models relevant for a specific case. This study focuses on clusters of microcalcifications and introduces an automated, flexible toolbox designed to generate microcalcification cluster models customized to specific tasks.

**Methods:**

The toolbox allows users to control a large number of simulation parameters related to model characteristics such as lesion size, calcification shape, or number of microcalcifications per cluster. This leads to the capability of creating models that range from regular to complex clusters. Based on the input parameters, which are either tuned manually or pre‐set for a specific clinical type, different sets of models can be simulated depending on the use case. Two lesion generation methods are described. The first method generates three‐dimensional microcalcification clusters models based on geometrical shapes and transformations. The second method creates two‐dimensional (2D) microcalcification cluster models for a specific 2D mammographic image. This novel method employs radiomics analysis to account for local textures, ensuring the simulated microcalcification cluster is appropriately integrated within the existing breast tissue. The toolbox is implemented in the Python language and can be conveniently run through a Jupyter Notebook interface, openly accessible at https://gitlab.kuleuven.be/medphysqa/deploy/breast‐calcifications. Validation studies performed by radiologists assessed the level of malignancy and realism of clusters tuned with specific parameters and inserted in mammographic images.

**Results:**

The flexibility of the toolbox with multiple simulation methods is illustrated, as well as the compatibility with different simulation frameworks and image types. The automation allows for the straightforward and fast generation of diverse microcalcification cluster models. The generated models are most likely applicable for various tasks as they can be configured in a variety of ways and inserted in different types of mammographic images of multiple acquisition systems. Validation studies confirmed the capacity to simulate realistic clusters and capture clinical properties when tuned with appropriate parameter settings.

**Conclusion:**

This simulation toolbox offers a flexible means of simulating microcalcification cluster models with potential use in both technical and clinical research in mammography imaging. The 3D generation methods allow for specifying many characteristics regarding the calcification shape and cluster architecture, and the 2D generation method presents a novel manner to create microcalcification clusters tailored to existing breast textures.

## 1 INTRODUCTION

Mammographic imaging is indispensable for breast imaging. Its use in screening plays a crucial role in early breast cancer detection,^1^ while diagnostic mammograms are essential for accurate diagnosis, treatment planning and follow‐up.^2^ Digital mammography (DM) acquires two‐dimensional (2D) images of a compressed breast in both a craniocaudal (CC) and a mediolateral oblique (MLO) view. More recent advances have extended this to (pseudo) three‐dimensional (3D) digital breast tomosynthesis (DBT) imaging. By reconstructing a limited series of projection images acquired over limited angular ranges,^3^, ^4^, ^5^ DBT aims to overcome the masking effect of lesions being potentially obscured by overlapping tissue in 2D imaging. Another technique is contrast‐enhanced mammography (CEM), which shows the uptake of iodinated contrast agent in neo‐vascular structures of suspicious regions.^6^ Despite these advances and improvements in image acquisition and processing,^7^ correctly interpreting mammographic images for a range of tasks, including, for example, risk assessment and diagnosis, remains challenging due to the diversity and potential subtlety of breast lesions.

Together with space‐occupying masses and architectural distortions, small deposits of calcium or “calcifications” are a common finding in the breast. While many calcifications are benign, some can be a sign of an underlying malignant process,^8^, ^9^ with approximately one‐third of breast cancers detected solely by the presence of microcalcification clusters.^10^ Whereas benign calcifications are typically larger and regular in shape, clusters of malignant calcifications often display a larger variation in morphology.^11^ Their wide variety in shape, size, and density can confound their detection and characterization. Efforts have been made to describe the features of microcalcification clusters, encompassing both shape and distribution. Le Gal et al.^12^ considered various configurations, and more recently, the fifth edition of the Breast Imaging‐Reporting and Data System (BI‐RADS) lexicon included a description according to morphology and distribution.^13^

Simulated models aim to represent the physical characteristics of real biological structures, serving many purposes in medical applications. In terms of modeling, breast lesion models of either masses or microcalcification clusters often consist of voxel volumes with assigned binary values, with “0” denoting the background and “1” indicating the lesion. In a recent review article, a variety of methods to simulate breast lesion models was described.^14^ Developing microcalcification cluster models is challenging due to the diversity in calcifications, and the variability in spatial distribution of single microcalcifications within clusters. A single model type cannot capture the broad spectrum of characteristics present in clinical cases. Furthermore, the study hypothesis or clinical application influences the required level of realism and modelled properties. While simpler models might be suitable for image processing optimization, realistic simulations are relevant for fine‐tuning diagnostic tools, enhancing medical training or advancing research in imaging techniques.

The present study seeks to build upon previous work^15^ by simulating extensive sets of microcalcification cluster models, taking into account a broader range of user requirements for the application at hand. We aggregate and develop several methods with adjustable parameters to simulate 3D microcalcification clusters of different clinical types and with different characteristics. In addition, we also provide a novel method to generate realistic microcalcification cluster models starting from the breast textures of 2D projection images,^16^ and aim to improve the realism of the final image containing the inserted calcifications. Both methods have been integrated into the automated toolbox described here, designed to streamline the simulation process and to provide the means to generate sets of microcalcification cluster models for clinical and research questions.

## 2 METHODS

We present both 3D and 2D methods for microcalcification cluster modeling, which are included in an automated toolbox that can generate databases of all model types. We explain how such models can be inserted into mammographic images with a hybrid simulation framework^17^ and finally report on the assessment of the realism of simulated clusters.

### 2.1 Simulation of 3D single calcifications and microcalcification clusters

#### 2.1.1 Simulation of 3D single calcifications from geometrical shapes

The first method generates single calcification models initialized from binary 3D geometrical shapes with specified dimensions in millimeters (mm). These can be spheres, ellipsoids,^15^ (elliptic) cylinders, and a specific “teacup” type. For this last case, two identical ellipsoids are created with one shifted along the z‐axis. Subtraction creates a teacup‐type calcification, similar to the method described by Näppi et al.^18^ Additionally, a random model shape type is available, which is not initialized from a central shape, but is solely composed of the noise values introduced in the next paragraph.

To create a more realistic calcification shape, irregular boundaries are introduced with the aid of uniformly distributed noise in the range of [0.0, 1.0]. After specifying a threshold noise level σ, the 3D array of the original geometrical shape is multiplied with an array of the same dimensions containing uniformly distributed noise values, and all values higher than σ are preserved as value “1,” whereas the others are set to “0.” Afterwards, the process is repeated for the initial shape with inverted voxel values and new noise values, now preserving all values above 1‐σ. Next, binary morphological opening operations are applied to remove small regions of connected voxels with “1” values within regions of “0” voxel values, as well as closing operations to fill holes of voxels with “0” values in regions with “1” voxel values. The largest volume of connected voxels is retained. Finally, it is possible to rotate the resulting calcification model in all three planes before returning it as an output of the generation method.

When generating single calcifications, information on the shape, final size, rotation, and sphericity is saved in a table. The sphericity is computed in Equation 1,

(1) $$\text{sphericity}=\frac{\pi^{1/3}{\left(6V\right)}^{2/3}}{A}$$

where the volume V is defined as the number of non‐zero voxels. Surface area A is approximated for a sphere, ellipsoid, random, or teacup type with dimensions x,y,z in Equation 2, and for an elliptic cylinder with radii x and y, and height z in Equation 3. Note that for cylinders, the sphericity is less meaningful on account of their elongated profile.

(2) $$\begin{array}{c} A=4\pi {\left(\frac{{\left(xy\right)}^{1.6}{\left(yz\right)}^{1.6}{\left(zx\right)}^{1.6}}{3}\right)}^{1/1.6} \end{array}$$

(3) $$\begin{array}{c} A=2\pi yz+2\pi \sqrt{\frac{y^{2}+z^{2}}{2}}x \end{array}$$

#### 2.1.2 Segmentation of 3D single calcifications from clusters

In addition to generating new 3D calcification models, existing 3D microcalcification cluster models can be re‐used through segmentation. Those can either be simulated clusters or real clinical clusters acquired through imaging. After rescaling the voxel intensity values within the imaged clusters to values in the range [0.0,1.0], a threshold of 0.5 is applied to the intensity to create a cluster model with binary voxel values. Each volume of connected “1” voxels is saved as a binary model of a single calcification. As with the geometrically generated models from Section 2.1.1, a table stores information regarding the size and shape of each calcification in the resulting database.

#### 2.1.3 Combination of single microcalcifications into 3D cluster models

There are two options to compile a cluster from calcifications: (i) geometrically generated calcifications are simulated on‐the‐fly with the techniques described in Section 2.1.1 and immediately added to a new cluster configuration; (ii) calcifications are selected based on size and sphericity from a provided database, which can either consist of previously geometrically generated calcifications or calcifications segmented from clusters as described in Section 2.1.2.

The method creates a cluster of designated volume and voxel size, with the placement of calcifications occurring either at predefined locations, on a structured grid, or randomly. In the latter case, a minimum distance between calcifications can be imposed. Calcifications are either simulated or selected from a database, and then added until the specified number is reached thus creating a new microcalcification cluster. During this process, calcifications can be rotated or, if necessary, resized to smaller or larger volumes (specified in mm), when insufficient calcifications of the required size are available.

As with the individual calcification models, this method provides the means to automatically simulate a large set of microcalcification cluster models and save them in a binary 3D format. A table stores relevant information on the clusters in the generated set, such as the model size, the included calcifications, and where those are placed in the cluster model.

### 2.2 Simulation of 2D microcalcification clusters from image‐specific tissue textures

The codebase also includes a method to simulate clusters considering the textures already present in the image. The method first establishes a plausible cluster location from a radiomics analysis of the whole breast and then determines potential locations of single calcifications from the local textures at this cluster location.^16^ This method has been developed for a dataset of 2D mammographic images with clinical image processing applied, referred by their DICOM attribute “Presentation Intent Type,” which is set to “For Presentation.” These images have an inverted lookup table in which calcifications show up as bright pixels with high attenuation. We show the application to pre‐processed images with the attribute set to “For Processing” is possible as well by inverting the pixel values prior to the processing steps. The final result consists of a binary 2D cluster model with value “0” for the background and values equal to “1” for the calcifications.

#### 2.2.1 Choice of location

An appropriate location for a microcalcification cluster in the breast region of a specific 2D mammographic image is first established. The procedure starts by segmenting the breast from the surrounding air, and then tiles the breast into a grid of non‐overlapping cells of chosen size,^19^ typically 200 × 200 pixels. In deciding upon the location, it is assumed that clusters develop more frequently in dense breast tissue regions or regions with more variety in structures. For every grid cell, a radiomics analysis^20^ performed with the PyRadiomics toolbox^21^ then computes 22 radiomics features chosen for their relevance to find such locations in the breast textures (see supplemental 1). For each feature the cells are sorted, either from highest to lowest feature value or vice versa depending on the feature, and the 10 top‐scoring cells are chosen. This results in a list of 22 × 10 grid cells from which the most prevalent cell is considered to be the optimal location. Users can choose to omit this step if they prefer to manually specify the center and size of the grid cell. The center of the cell is not always equal to the center of the simulated cluster due to the choice of calcifications within the cell in the following steps.

#### 2.2.2 Choice of calcifications from local textures

**FIGURE 1:**
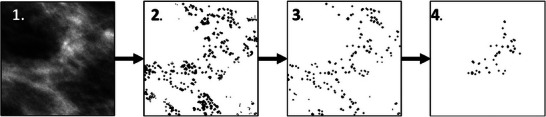
Pipeline to create a cluster from local textures: step 1) choose a grid cell; step 2) create a mask with candidate calcifications; step 3) filter candidate calcifications for size and circularity; step 4) build a cluster starting at the location with highest contrast.

For the selected cell, a microcalcification cluster is created from candidate calcifications as shown in Figure 1. Possible locations of single calcifications within the selected cell are found by combining two masks that consider local textures in the breast. A first mask of the selected cell is obtained by saving all pixels with an intensity value above the median, since it is assumed the calcifications will then blend in more smoothly with the surrounding pixels, as they also exhibit high pixel intensity. A second mask is obtained by applying a Frangi filter,^22^ to detect regions that already show more structure with a variety in pixel values. Again, it is assumed that this will improve the realism of the calcifications since they exhibit a discernible structure with somewhat sharp boundaries. The multiplication of the intensity mask and the Frangi mask results in a final binary mask of the same size as the selected cell, with each region of connected “1's” denoting a candidate calcification.

In the last step, only calcifications within specified ranges of size and circularity are kept. The latter is computed by Equation 4, with the surface area A being equal to the number of “1's”, and the perimeter P=2πmax(x,y) for radii x and y.

(4) $$\begin{array}{c} circularity=\frac{4\pi A}{P^{2}}\; \end{array}$$

In order to build a cluster from these candidate calcifications, the contrast in the selected cell of the image is computed within an overlapping grid of smaller cells of 10 × 10 pixels. The equation implemented in the PyRadiomics toolbox^21^ (Equation 5) considers the pixel values (PV) of all pixels (i,j) within the cell.

(5) $$contrast=\sum_{i=1}^{10}\sum_{j=1}^{10}{\left(i-j\right)}^{2}PV\left(i,j\right)$$

Cluster creation starts within an initial search region of the sub‐cell with the highest contrast that includes at least one (part of a) calcification. Each candidate calcification within this region is added to the cluster. An iterative process of enlarging the search region around the previously included calcifications then incorporates additional calcifications until the specified number is reached. This results in a binary 2D model of a microcalcification cluster tailored to the existing textures of the breast in the specific image.

### 2.3 An automated toolbox

**FIGURE 2:**
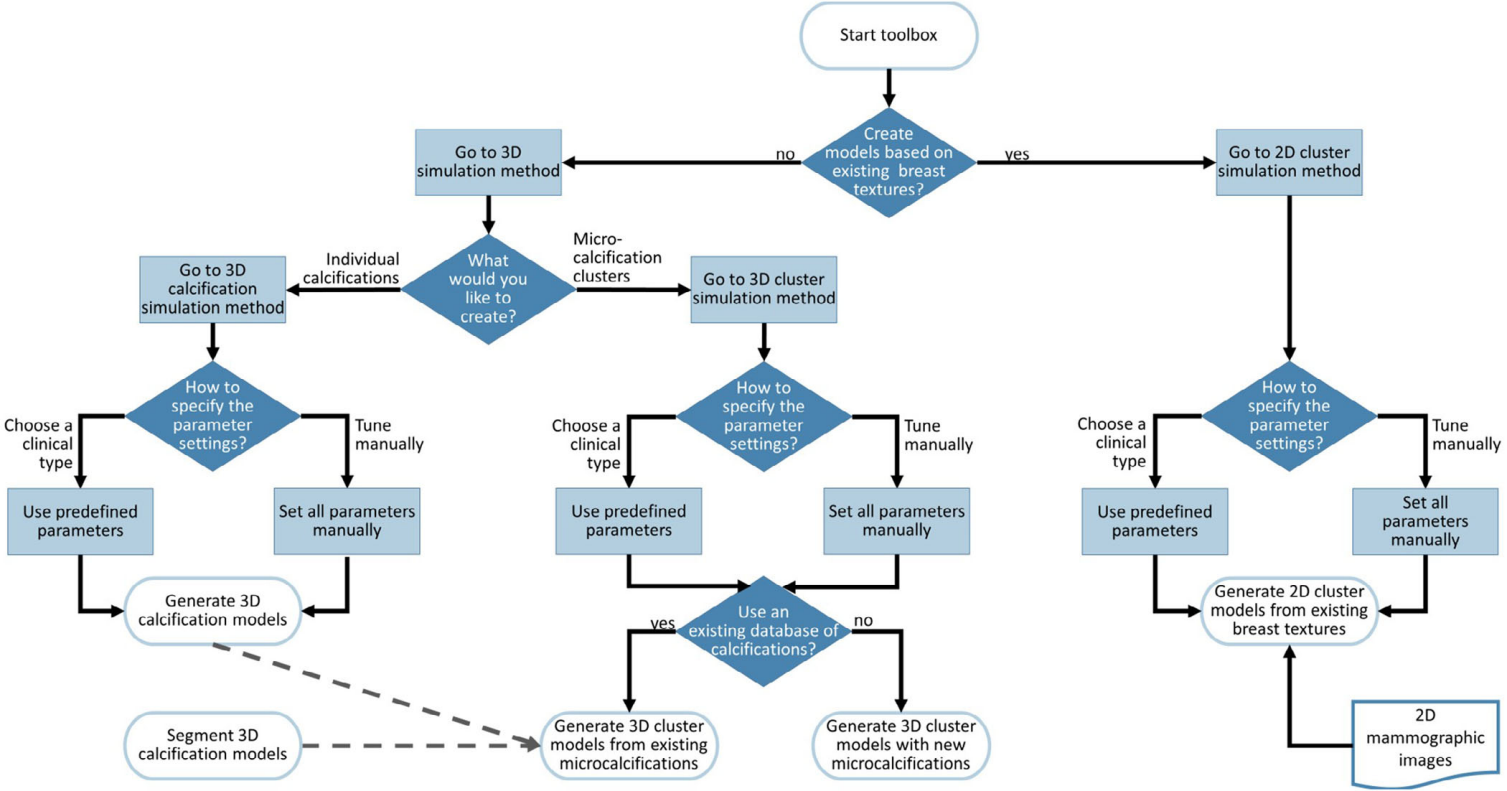
Structure of the toolbox to determine the parameter settings and generate models.

We have integrated the modeling methods into a toolbox that can be used to automatically create databases of microcalcification cluster models. This is accessible through a Jupyter notebook interface at https://gitlab.kuleuven.be/medphysqa/deploy/breast‐calcifications, which supports different operating systems. Modeling parameters are collected via a tree‐like set of questions shown in Figure 2. In the first section, the users specify whether 3D models or rather 2D models customized to the breast textures will be simulated. A next question inquires how the parameters of the simulation should be set, either manually or based on the clinical type.

If the user has opted to generate 3D microcalcification cluster models from an existing database of individual calcifications, this database has to be uploaded. For the 2D models, on the other hand, a table containing the filenames of the images in which to insert a microcalcification cluster is required. The insertion location can either be selected automatically with the methods from Section 2.2.1, or pre‐set by the user in which case this location should be defined in the table. This enables the automatic creation of new cluster models tailored to the breast textures in each image in the data table.

#### 2.3.1 Tune simulation parameters manually

In the first option for parameter setting, values are adjusted manually in the toolbox. For the 3D simulations, Table 1 represents all the parameters that can be tuned. These values can either be selected from a predefined list of options or chosen from a continuous range. For all continuous parameters except the voxel size, both a minimum and a maximum value should be selected. The toolbox then selects random values from within the ranges for each model that will be added to the database. When calcifications within a cluster are placed on a grid, the minimum distance is the exact distance between calcifications, whereas for randomly placed calcifications, it is only a lower boundary. When the user opts to place calcifications at predefined locations, an additional file containing a list of these locations within the cluster model should be provided.

**TABLE 1:** Tuning parameters for the 3D models.

Parameter	Tuning method	Options
Shape calcifications	Selection	Spherica Ellipsoidal Cylindrical Teacup Random
Size calcifications (in three dimensions)	Range	0.01 –2.00 mm (step 0.01 mm)
Sphericity calcifications	Range	0.01–2.00 (step 0.01)
Rotation angle calcifications	Range	0°–360° (step 1°)
Threshold noise value	Range	0.00–1.00 (step 0.01)
Voxel size	Range	0.000 –1.000 mm (step 0.005 mm)
Size clusters (in three dimensions)	Range	0.10 –100.00 mm (step 0.01 mm)
Number of calcifications	Range	1–100 (step 1)
Placement of calcifications in cluster	Selection	Random Grid Predefined

Similarly as for the 3D models, Table 2 provides an overview of parameters that can be tuned for the 2D microcalcification cluster models. This list is short since the method to generate image‐specific clusters already considers existing breast textures to tune calcification shapes and cluster architectures, and thus allows less freedom in manual tuning.

**TABLE 2:** Tuning parameters for the 2D models.

Parameter	Tuning method	Options
Size calcifications	Range	0.01 –2.00 mm (step 0.01 mm)
Circularity calcifications	Range	0.01–2.0 (step 0.01)
Number of calcifications	Range	1–100 (step 1)

#### 2.3.2 Use of pre‐defined parameters for a specific clinical type

In the second option for parameter setting, regarding the clinical type, different cluster morphologies for the 3D models can be chosen based on the BI‐RADS categories.^13^ Since the 3D models do not consider any surrounding tissue, BI‐RADS categories which heavily depend on existing breast structures, such as vascular, skin and suture calcifications, are not included. From the included categories, four are typically benign: round, punctate, milk of calcium, and large rod‐like; and four are of suspicious morphology: amorphous, coarse heterogeneous, fine pleomorphic, and fine linear (branching). Supplemental 2 lists the set of parameters for individual calcifications and microcalcification cluster models, pre‐determined based on descriptions from literature^11^, ^13^, ^23^ and visual inspection.

Similarly, the toolbox differentiates between typically benign clusters and clusters of suspicious morphology for the 2D models. For each category, a fixed set of parameters has been implemented in the code. Further validation of this differentiation is discussed in Section 2.5.

#### 2.3.3 System requirements

When developing the toolbox, no specific system requirements or limitations were taken into account. All simulations described in this work were performed with an Intel Core i7 – 10850H central processing unit (CPU) and 16 Gigabyte (GB) of random access memory (RAM). Other CPUs or devices might encounter longer simulation times than those described in the results section.

As an example, we generated sets of 10 clusters for each of the specific BI‐RADS categories of the 3D models, and considered the time and memory requirements. Generating and storing the 3D models is demanding in terms of required memory, due to the large size of the cluster in mm combined with the small voxel size required to simulate microcalcifications. Therefore, a method to save the models in a sparse format (NPZ) without any loss of information is included, which reduces computation time and memory requirements. In the toolbox, the user can then opt whether to use this sparsity, or save models in a full RAW format. For the 10 BI‐RADS categories, we discuss the results of both options.

### 2.4 The insertion of generated models into clinical mammographic images

With the implemented toolbox, it is possible to create databases of microcalcification cluster models. Verification of realism, correctness, and appropriateness of the models for use in applications, requires their insertion into mammographic images. Several methods exist to combine the calcification models with the background image.^14^ With intensity scaling methods, the intensity and contrast of the lesion models are adapted to match the background image. In addition, total simulation frameworks replace normal tissue of a 3D simulated breast with the lesion model prior to generating the projection images.^24^, ^25^, ^26^ An alternative method is to use a hybrid framework in which lesions are simulated directly into a projection image,^27^, ^28^ typically of real breasts.

To this end, we illustrate the insertion of the generated models in mammographic images of available DM, DBT, and CEM datasets. Among the existing frameworks, we chose an existing hybrid simulation framework^17^ to create the illustrative results. This framework was converted to Python and extended in terms of functionality and the number of different acquisition systems modeled. It facilitates the insertion of binary voxel models of breast lesions into existing mammographic images at a specified location, which could be either defined manually or computed with the methods described in Section 2.2.1. For any 3D binary voxel model of a lesion, this involves four main processing steps: (1) generate or import the binary voxel model, (2) ray‐trace to generate an ideal 2D template of the projection of the type “exp(−μT),” with μ being the x‐ray linear attenuation coefficient of a breast lesion with thickness T, (3) incorporate degrading factors such as x‐ray scatter and blurring, and (4) insert the modified template into the original image by multiplication. This complete four‐step process can be applied to all types of 3D binary cluster models generated in Section 2.1. Modifications to generate a template for the 2D microcalcification cluster models of Section 2.2 are detailed in Section 2.4.2.

This hybrid framework was designed for insertion into “For Processing” images, in which the x‐ray signal intensity is proportional to the x‐ray energy absorbed in the x‐ray detector, with low pixel values indicating a low signal level in the detector and vice versa. Ray‐tracing of a binary voxel model of a lesion thus results in a normalized projection template with real template value (TVs) ranging between 0.0 and 1.0. At calcification locations TVs lower than 1.0 reduce signal intensity after multiplication, whereas at background locations, TVs equal to 1.0 do not alter the original intensity. Modifications required to generate templates suitable for “For Presentation” images are described in the next section.

#### 2.4.1 Modifications to generate templates for “For Presentation” images

Inserting lesions in “For Presentation” images requires a modification, as these images use an inverted lookup table in which high attenuation is shown with high (bright) pixel values and the contrast of calcifications is no longer determined by the attenuation coefficients. An additional processing step was included to modify TVs representing calcifications before the multiplication. To this end, an empirical relationship was determined between the contrasts of real calcifications in “For Processing” images and the same calcifications in “For Presentation” images. For each calcification, the pixel intensities in equivalent areas of calcification and adjacent background regions were measured. The Weber contrast between the mean pixel values (MPVs) of these paired regions was calculated in “For Processing” and “For Presentation” images using Equation 6.

(6) $$contrast=\frac{MPV_{calcification}-MPV_{background}}{MPV_{background}}$$

For each acquisition system investigated (GEHC Senographe Essential, GEHC Senographe Pristina, Siemens Mammomat Inspiration and Siemens Mammomat Revelation), the quasi‐linear relationship of Equation (7) was found, resulting in system‐specific fit coefficients $w_{contrast}$ and $k_{contrast}$.

(7) $$\begin{array}{ccc} & & \;contrast_{For\;Presentation}=w_{contrast}\;*\\ & & contrast_{For\;Processing}+k_{contrast} \end{array}$$

The first three steps of the simulation framework generate a modified 2D template for a “For Processing” image. To modify the template for “For Presentation” images, the calcification TVs are corrected with the measured fit coefficients of Equation (7). In the resulting template, regions of high attenuation, that is, calcifications have values greater than 1.0. When the template is multiplied into the mammographic image, the pixel intensities of regions containing the calcification are increased, giving the bright calcifications seen in images.

#### 2.4.2 Modifications for inserting 2D cluster models

As ray‐tracing of a 2D model cannot be performed, the insertion of clusters from Section 2.2 in a mammographic image requires some additional modifications to the simulation process. An intensity scaling method was developed, which aimed to approximate a template similar to the one generated in step 2) of the hybrid simulation framework of Section 2.4 for an existing binary 2D cluster model. This method consisted of two steps: (1) apply a distance transform to the 2D model, and (2) use an empirically established relationship to approximate the ray‐traced TVs, comprising three weighting factors: a distance transform weighting $w_{dist}$, a breast thickness weight $w_{thick}$, and a tube voltage weight $w_{volt}$.

In order to determine the weighting factors, 3D binary voxel models of aluminum spheres of diameters 0.1–1.0 mm, assumed to represent calcifications, were utilized. These spheres were ray‐traced with the hybrid simulation framework, for backgrounds of different thicknesses, and for all investigated acquisition systems. The resulting sphere templates had 1.0 values in the background and minimum values at the center of the sphere, which were lower for larger spheres.

In parallel, the sphere templates were converted to have binary values of “0” in the background and “1” in the sphere, such that they could be considered equal to a simple 2D cluster model with a single spherical calcification. A distance transform was then used to compute the Euclidean distance to the nearest background location for every location in this simplified 2D model, and saved this distance as the respective value.

Next, the values in the ray‐traced sphere template were related to the values after the distance transform by applying a linear regression to find $w_{dist}$ and intercept $k_{intensity}$. Additionally, factors for the background thickness and tube voltage for the specific background and acquisition system were included with weights $w_{thick}$ and $w_{volt}$, respectively. Altogether, the computed weights and intercept were combined in Equation 8 to describe the TVs of the ray‐traced sphere in relation to those obtained after distance‐transform.

(8) $$\begin{array}{ccc} & & \;TV_{ray\_traced}=w_{dist}\;*TV_{distance\_transformed}\\ & & +w_{thick}*thickness+w_{volt}*voltage+k_{intensity`} \end{array}$$

The established intensity scaling method to create a template of the 2D cluster model, as if it was ray‐traced, thus combines both steps. It first applies the distance transform to the binary values of the cluster model, resulting in a template with background values of 0.0 and calcification values equal to the distance to the background. Next, the non‐zero calcification values are adjusted with Equation 8 using image‐ and system‐specific weights. After setting all background TVs equal to 1.0, a Gaussian filter is applied to smooth calcification edges.

As this process replaces the steps of (1) lesion generation and (2) ray‐tracing, the resulting template is comparable to an ideal 2D template generated by the simulation framework with TVs approximating the exp(−μT) projection. Steps (3) incorporating degrading factors and (4) insertion into the image can be performed without further modification. Similarly as the original pipeline of the hybrid simulation framework, the resulting template is applicable for insertion in “For processing” images. In order to insert in a “For Presentation” image, the procedure described in Section 2.4.1 should be applied between steps (3) and (4).

### 2.5 Assessment of the realism of simulated lesions

For certain applications, simulated lesions should have a realistic appearance. This can be the case for deep learning model training or certain virtual clinical trials (VCTs). Therefore, this section briefly describes two separate, but comparable validation studies similar to earlier described work^28^, ^29^ to assess the realism of real and simulated clusters generated with specific parameter settings.

In previous work for the 3D models,^15^ simulation parameters were manually chosen for benign clusters based on findings in the literature.^30^, ^31^, ^32^ This resulted in spherical calcifications of diameter 0.09 –0.6 mm, and between 8 and 25 of these calcifications were added to cubic clusters ranging from 2.25  to 6.00 mm in extent. For the malignant case, single calcifications were segmented from real clusters with the method of Section 2.1.2. These were added to clusters of the same size as the benign clusters, but containing between 18 and 35 calcifications. The hybrid simulation framework was then used to insert the 3D cluster models into low‐energy images of the CEM dataset described by Beuque et al.^33^ The insertions were performed at locations with high skew.^15^

Of the 40 real cases and 40 cases with simulated clusters, half were shown with one view only (either CC or MLO) and for the other half both views were shown. In this work, we consider the results for one view. Two radiologists (with three and nine years of experience in breast imaging) scored each case, blinded to the outcomes. The readers were asked to answer the first question, “How confident are you this cluster is realistic?”, using a six‐level confidence scale with possible answers 1: “Extremely confident cluster is simulated”; 2: “Moderately confident cluster is simulated”; 3: “Slightly confident cluster is simulated”; 4: “Slightly confident cluster is real”; 5: “Moderately confident cluster is real”; and 6: “Extremely confident cluster is real.” Similarly for the second question, “How confident are you this cluster is malignant?”, answers were given on a six‐level confidence scale with possible answers 1: “Extremely confident cluster is benign”; 2: “Moderately confident cluster is benign”; 3: “Slightly confident cluster is benign”; 4: “Slightly confident cluster is malignant”; 5: “Moderately confident cluster is malignant”; and 6: “Extremely confident cluster is malignant.”

To validate the 2D models, these were simulated for specific low‐energy images of the same dataset. After choosing an optimal location with the method from Section 2.1.2, and simulating 20 “typically benign” cases and 20 cases of “suspicious morphology” discussed in Section 2.3.2, they were combined with 40 real cases. Again, one radiologist experienced in mammography rated the realism and level of malignancy on the same scales under the same conditions.

A Mann‐Whitney *U*‐test was applied to compute the *p*‐values comparing the results of different subsets (i.e., real benign clusters, real malignant clusters, simulated benign clusters, and simulated malignant clusters). A *p*‐value of 0.05 was set to be the limit for significant differences. In addition, receiver operating characteristic curves (ROCs) were computed to study the discrimination between real and simulated clusters. When the area under this curve (AUC) is close to 1.0, it means a high level of discrimination between two sets (e.g., benign vs. malignant, or real vs. simulated) can be obtained, whereas values close to 0.5 denote the guessing level.

## 3 RESULTS

This section provides an overview of the possibilities and restrictions when modeling microcalcification clusters with the automated toolbox by illustrating some examples of the resulting images with simulated clusters inserted.

### 3.1 Variety in the simulated 3D microcalcification cluster models

**FIGURE 3:**
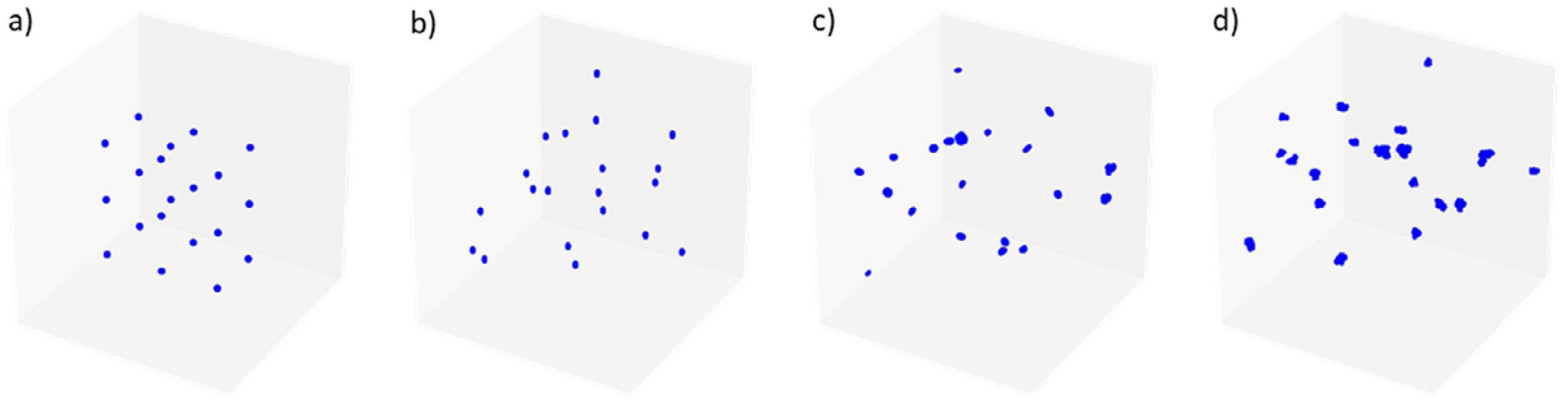
Four generated 3D microcalcification cluster models with versatility of calcifications increasing from left to right. All clusters are 30 × 30 × 30  mm and contain 20 microcalcifications.

The method to create 3D microcalcification cluster models described in Section 2.1 enables manual parameter tuning that can produce a wide variety of clusters. As an illustration, the four clusters in Figure 3 all have the same size and number of microcalcifications, but different morphological characteristics. Whereas the calcifications in cluster (a) are all spherical and placed on a regular grid, the others include more variety. Cluster (b) consists of ellipsoidal calcifications, all of which have the same dimensions with a low noise level threshold equal to 0.05, placed randomly in the cluster volume. More variety is introduced in cluster c() by allowing rotations and a higher noise level threshold as well as defining a range from which the dimensions are chosen. For the most irregular cluster (d), randomly shaped calcifications are created with a higher noise threshold and rotations.

**FIGURE 4:**
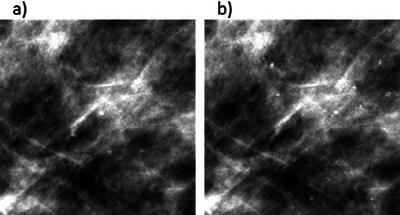
Two examples of generated 3D microcalcification cluster models of a specific clinical type inserted in a 2D mammographic “For presentation” image: (a) shows a milk‐of‐calcium‐type cluster; and (b) a coarse heterogeneous‐type cluster.

In addition to tuning parameters manually, the 3D generation method can create models of specific BI‐RADS types. As an illustration, Figure 4 shows (a) a milk‐of‐calcium‐type cluster, and (b) a coarse heterogeneous‐type cluster on the right, both inserted at the same location in the same breast.

### 3.2 Variety in the simulated 2D microcalcification cluster models

Similarly as for the 3D generation method, 2D microcalcification cluster models can be created by either tuning parameters manually or by choosing a specific clinical type. Due to the restrictions already implied by modeling the cluster to fit existing breast textures, only a limited number of cluster characteristics such as size and circularity, can be imposed by the user.

**FIGURE 5:**
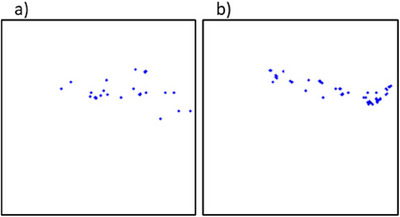
A pair of generated 2D microcalcification cluster models with low variety for (a), and more variety for (b). Both clusters are obtained for the same location in the same mammographic image.

Figure 5 shows a pair of 2D models with both clusters simulated for the same location in the same mammographic image. In (a) small ranges in calcification shape and size were permitted, whereas (b) allowed for larger ranges of calcification circularity and size.

**FIGURE 6:**
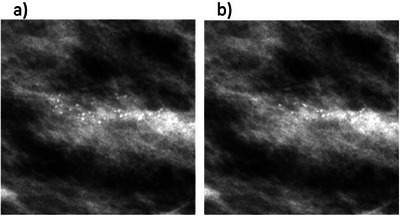
Two examples of generated 2D microcalcification cluster models of a specific clinical type inserted in a 2D mammographic “For presentation” image: (a) a typically benign cluster; and (b) a cluster of suspicious morphology.

Figure 6 shows an example of each clinical type available in the 2D simulation method. Figure 6a consists of a typically benign cluster and Figure 6b consists of a cluster of suspicious morphology, customized for the same location in the same image.

### 3.3 An automated toolbox

The previous sections show that the toolbox and its adjustable parameters can generate a variety of microcalcification cluster models. In theory, a set of models can be of any size although system storage capacity will ultimately limit the set size. Since the user can specify minimum and maximum values for ranges for many of the simulation parameters, most of the generated clusters will be unique due to the random sampling of a value within the range for each specific simulation.

To illustrate the time and memory requirements, 3D models of all included BI‐RADS types were generated. The results can be found in Table 3. For the amorphous and fine linear clusters only the sparse model is considered, since the full models were too large to store. For all other categories, the full models typically require 7.0–10.0 GB of memory, whereas this significantly drops to about 350 Megabyte (MB) for the sparse models. Regarding the average time to create a model, there are no large differences between the full models and the sparse models, neither for single calcifications nor for clusters.

**TABLE 3:** Time and memory requirements for the simulation of 3D models.

		Full models			Sparse models		
BI‐RADS type	Voxelsize (mm)	Average time calcification simulation (s)	Average time cluster simulation (s)	Maximum memory usage cluster simulation	Average time calcification simulation (s)	Average time cluster simulation (s)	Maximum memory usage cluster simulation
Round	0.01	1.77	14.72	7.0 GB	1.42	16.00	365 MB
Punctate	0.01	0.25	8.62	9.6 GB	0.34	4.55	343 MB
Milk of calcium	0.01	2.58	32.75	9.9 GB	2.56	26.04	330 MB
Large rod‐like	0.05	0.09	1.13	60 MB	0.13	1.31	60 MB
Amorphous	0.005	0.12			0.10	7.13	119 MB
Fine pleomorphic	0.01	0.94	29.50	10.6 GB	1.04	28.07	356 MB
Coarse heterogeneous	0.01	0.16	19.30	10.6 GB	0.13	9.30	223 MB
Fine linear	0.005	4.80			8.70	138.08	2.3 GB

### 3.4 The insertion of simulated models in clinical mammographic images

**FIGURE 7:**
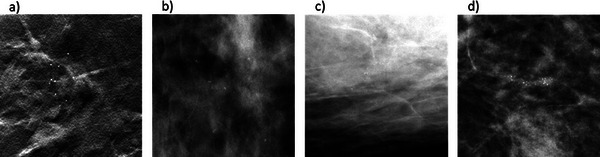
Four examples of generated models inserted in different types of mammographic images. (a) A reconstructed slice of a 3D punctate cluster inserted in a “For Processing” DBT image acquired on a Siemens MAMMOMAT Revelation system; (b) A 3D pleomorphic cluster inserted in a phantom breast and projected by the VICTRE platform to obtain a DM image; (c) A 2D cluster of suspicious morphology in a “For Processing” DM image acquired on a GEHC Essential system; (d) A 2D cluster of suspicious morphology in a “For Presentation” DM image acquired on a Siemens MAMMOMAT Inspiration system.

The toolbox creates lesion models for use with any simulation framework and into any type of projection image of the breast. As an example, a simulated lesion was inserted in DBT projection images using our in house simulation framework,^34^ and Figure 7a shows one of the reconstructed DBT slices.

Another successful example of lesion insertion is obtained with the VICTRE in silico imaging pipeline,^26^ which produced the simulation in Figure 7b to illustrate the compatibility with other simulation frameworks. A 3D model was inserted in a digital phantom to obtain a simulated DM image. Since the sparse matrices in NPZ format can be difficult to handle by software tools other than Python, the model was converted to RAW format before insertion.

The applicability of the 2D texture‐based cluster generation method is illustrated as well. Despite differences in pixel values and image processing in general between acquisition systems, the resulting feature values still return appropriate locations and plausible microcalcifications. Figure 7c and d show a 2D cluster simulated for a “For Processing” image acquired on a GEHC Senographe Essential system and for a “For Presentation” image acquired on a Siemens MAMMOMAT Inspiration system, respectively.

### 3.5 Assessment of the realism of simulated lesions in validation studies

**FIGURE 8:**
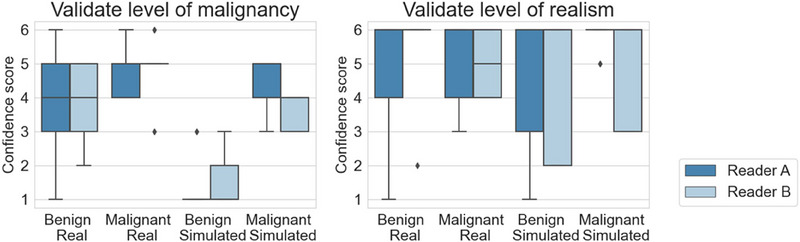
Results of a validation study of images with 3D microcalcification cluster models inserted. Scores ranged from 1: “most probably benign” to 6: “most probably malignant” for the assessment of the level of malignancy, and from 1: “extremely confident simulated” to 6: “extremely confident real” for the assessment of the realism.

The validation results of 3D models are shown in Figure 8. The left subfigure shows that simulation parameters were selected in such a way that radiologists could clearly distinguish between benign and malignant simulated clusters. They had more difficulty in making this distinction for real cases with both the real benign and real malignant clusters receiving an average score of 4 or higher. Simulated benign clusters had probably many specifically benign characteristics, resulting in a lower level of realism than for the real benign clusters. The realism scores attributed to real and simulated malignant clusters in the right subfigure were both high. All of the statistical results (radiologist A: *p* = 0.58, AUC = 0.62 for benign, *p*  = 0.44, AUC = 0.36 for malignant; radiologist B: *p* = 0.60, AUC = 0.60 for benign, *p* = 1.00, AUC = 0.52 for malignant) denote there was no significant perceived difference between the distribution of realism scores obtained for either real or simulated clusters.

**FIGURE 9:**
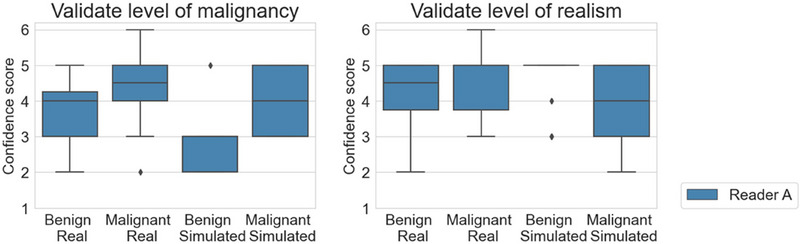
Results of a validation study of images with 2D microcalcification cluster models inserted. Scores ranged from 1: “most probably benign” to 6: “most probably malignant” for the assessment of the level of malignancy, and from 1: “extremely confident simulated” to 6: “extremely confident real” for the assessment of the realism.

The plots in Figure 9 depict the results of the validation study with simulated 2D models. The realism scores in the right subfigure of simulated clusters were comparable to those given to the real clusters. The *p*‐value of 0.09 (AUC = 0.64) for malignant lesions signifies no significant perceived difference between real and simulated lesions, whereas the *p*‐value of 0.03 shows that there is one for benign lesions. However, the AUC value of less than 0.5 (AUC = 0.33), together with the larger mean score for simulated clusters suggests that the simulated benign clusters were considered more realistic than the real cases. For each category, the majority of cases received a score of 4 or higher, denoting the realistic appearance of the simulated microcalcification clusters.

Regarding the malignancy classification, all simulated benign cases except one received a score of three or lower. Scores for the malignant simulated clusters ranged from 3 to 5. Even though this range is higher than for the benign cases, not all were thus correctly classified. The same remark applies for the real categories, showing the difficulty to correctly classify microcalcification clusters based on mammographic images alone.

## 4 DISCUSSION

### 4.1 A novel microcalcification cluster modeling approach

The presented toolbox facilitates the generation of extensive simulated microcalcification cluster databases, with the option to customize parameter settings for varied shapes and configurations. The 3D simulation method offers increased flexibility in modeling the cluster characteristics, while the 2D simulation method ensures integration with the surrounding breast textures. The results illustrate the versatility of the tool, and the compatibility with different simulation frameworks and mammographic image types. Appropriate tuning or the use of pre‐determined values result in cluster models with a realistic appearance, confirmed by validation studies.

To our knowledge, our automated toolbox is the first to encompass such a wide range of microcalcification cluster modeling techniques. In previous work, a commonly used approach consisted of modeling a geometrical shape that was then altered in a variety of ways to create calcifications.^35^, ^36^ These methods only obtained a limited diversity, which the use of more advanced algorithms could partially overcome,^37^, ^38^ although they still focused on a single initial shape. In comparison, our approach to encompass multiple geometrical shapes, and to include noise and random values, overcomes the restriction of limited model variation and ensures unique models can be generated.

Other work increased the diversity and realism of generated models by the segmentation of real microcalcification clusters from either 2D^29^, ^39^, ^40^ or 3D^28^, ^41^ clinical images. When modeling new lesions, however, they remained constricted by the number of real segmented cases available. Carton et al.^42^ created new calcifications based on a principal component analysis (PCA) of the calcification model characteristics. PCA was used to characterize the main modes of variation in the 3D shapes by considering the geometrical transformations of one calcification to another. Large sets of realistic microcalcification cluster models could thus be created by reconstructing them from the PCA components, however without association with a clinical type or description. The link between specific clinical types and pre‐determined parameter values in this work aimed to bridge this gap between calcifications that are either realistic but with unknown clinical type,^28^, ^42^ or of a specific, yet less realistic shape.^18^, ^35^, ^43^ The limitation of the number of real cases available encountered in other studies^28^, ^41^ is overcome by segmenting calcifications and recombining them in new cluster configurations.

The technique to model 2D microcalcification clusters specific to existing breast textures is novel in the field. Plourde et al.^44^ described a method to grow microcalcifications considering the pixel values of the surrounding background tissue. With the handcrafted radiomics analysis from Section 2.2, more features are considered for finding plausible insertion locations and candidate calcifications.

### 4.2 Limitations

Despite the capability to simulate a wide variety of shapes and distributions, the 3D models remain constrained by the geometrical properties and operations performed. A trade‐off exists between extensive manual parameter tuning to generate specific shapes and maintaining a level of randomness for realistic simulations. Some parameter combinations may yield unrealistic or non‐existing models, especially for small calcifications that require a small voxel size.

Generating binary lesion models introduces another challenge: an accurate simulation framework that accounts for acquisition system characteristics is required in order to obtain realistic images. This poses limitations on the type and origin of images for which simulations can be obtained. Application to a new imaging device would require the characterization of different physical parameters, and the framework should be correctly implemented and thoroughly validated for the specific device. A hybrid simulation framework can complicate the integration of the lesion model with existing breast textures, a limitation we tried to overcome by generating 2D models tailored to a specific image. The tendency to search for denser regions or regions with more structure, could inadvertently focus on, for example, the pectoral muscle or skin folds instead of on the breast tissue itself. An accurate initial breast mask is therefore necessary for realistic simulations. Furthermore, creating a case with the same lesion inserted in CC and MLO view still presents a challenge due to the difficulty in finding an architectural and positional correlation between both views.

Currently, the realism assessment was performed by two radiologists on images of one specific dataset. Comprehensive validation, which was not feasible within this study, would demand similar studies to be performed with images of all major vendors, all image types, and more radiologists. We have restricted ourselves to illustrating a few cases and showing how realistic images can be obtained with the generated models and adequate insertion techniques. In our validation study, it was also found that the radiologists had difficulties in accurately differentiating between real benign and malignant clusters. This shows the difficulties in correctly defining the characteristics that render a microcalcification cluster more suspicious and should be considered when developing modeling methods. Furthermore, the assessment of the clusters generated for specific clinical types (Section 2.3.2) only considered descriptors from literature and visual inspection. An accurate verification would require the insertion in magnification images of DM (or DBT), which was not feasible with the current simulation framework. As for the realism validation, we therefore encourage users to properly validate their generated models as well as their complete simulation pipeline for their specific application.

### 4.3 Applications and future prospects

The flexibility of the methods suggests a broad applicability for simulated clusters. The realistic appearance of mammographic images with simulated clusters configured with appropriate parameter values suggest potential application in medical training.^45^ These can illustrate specific conditions, such as difficult‐to‐detect calcifications.

Simulated breast lesion models are essential in VCTs,^14^ designed to model real clinical situations to investigate a specific task without the need to gather patient data.^46^ The type of simulated lesions varies depending on the task at hand. Diagnostic tasks or detectability studies benefit from more realistic microcalcification clusters, and to this end, radiologists can rate the realism.^28^, ^29^, ^38^, ^41^ Conversely, system parameter optimization might prefer regular clusters with certainty about the shape and configuration. As the 3D method is not image‐specific, nor system‐ or vendor‐specific, the same cluster model inserted into images of different acquisitions allows for side‐by‐side comparisons.

Furthermore, realistic simulations can overcome data scarcity. The generation of specific sets with simulated lesions should enable algorithm testing and deep learning model improvement for the detection and characterization of microcalcification clusters.^36^ Unlike deep learning techniques such as generative adversarial networks or diffusion models, our pipeline provides explicit modeling and transparency of the simulation process.

The future prospects involve deploying the developed toolbox and methods for insertion for these practical applications, in particular, deep learning model training and algorithm testing. This can include a comparison with models developed with other modeling techniques.

Whereas the current methodologies to create image‐specific clusters are only implemented for 2D mammographic images, this can be further extended for 3D modalities such as DBT. More spatial information could be taken into account in order to improve the architecture of the generated clusters and possibly their realism.

## 5 CONCLUSION

The toolbox introduced in this work provides a novel, automated and flexible method to simulate microcalcification cluster models that capture a large part of radiological reality. In addition to 3D calcification models, we also presented a method to simulate 2D microcalcification cluster models that are directly integrated within breast textures using radiomics features and image processing. Tuning the different simulation parameters creates models tailored to different needs. Cluster models with simple spherical calcifications could be beneficial in optimization studies, whereas more complex, realistic models can be provided also for deep learning model training and VCTs. Validation studies have proven the realistic appearance for several types of simulated clusters.

## CONFLICT OF INTEREST STATEMENT

Henry C. Woodruff: no disclosures related to the current manuscript. Outside of the current manuscript: Henry C. Woodruff has minority shares in Oncoradiomics SA. Philippe Lambin: no disclosures related to the current manuscript. Outside of current manuscript: Philippe Lambin has received grants and sponsored research agreements from Radiomics SA, Convert Pharmaceuticals SA, and LivingMed Biotech srl. He received presenter fees (in cash or in kind) and/or reimbursement of travel costs or consultancy fees (in cash or in kind) from AstraZeneca, BHV srl, and Roche. Philippe Lambin holds or held minority shares in Radiomics SA, Convert Pharmaceuticals SA, Comunicare SA, LivingMed Biotech srl, and Bactam srl. Philippe Lambin is a co‐inventor on two issued patents with royalties on radiomics (PCT/NL2014/050248 and PCT/NL2014/050728), licensed to Radiomics SA. One issued patent on mtDNA (PCT/EP2014/059089), licensed to ptTheragnostic/DNAmito. One granted patent on LSRT (PCT/P126537PC00, US Patent No. 12,102,842), licensed to Varian. One issued patent on the radiomic signature of hypoxia (U.S. Patent No. 11,972,867), licensed to a commercial entity. One issued patent on prodrugs (WO2019EP64112) without royalties. One non‐issued, non‐licensed patent on deep learning‐radiomics (N2024889). Three non‐patented inventions (software), licensed to ptTheragnostic/DNAmito, Radiomics SA, and Health Innovation Ventures. Philippe Lambin confirms that none of the above entities were involved in the preparation of this study. Hilde Bosmans: no disclosures related to the current manuscript. Outside of the current manuscript: Hilde Bosmans has sponsored research from Siemens healthineers and GE Healthcare. Hilde Bosmans is co‐founder and shareholder of Qaelum NV and Qaelum Inc. The other authors have no relevant conflicts of interest to disclose.

## Supporting information



Supporting Information

Supporting Information

Supporting Information

Supporting Information

## Data Availability

All code implemented and used for this paper can be found at https://gitlab.kuleuven.be/medphysqa/deploy/breast‐calcifications. Upon request generated databases of simulated models could be shared.
