# Supplementary material for: An automated toolbox for microcalcification cluster modeling for mammographic imaging
Source: Med Phys. 2024 Nov 21;52(2):1335–49. doi: 10.1002/mp.17521 (PMC11788264; doi:10.1002/mp.17521)
Supplement: Supplementary file 1 — Supporting Information [file MP-52-1335-s002.doc]

Supplemental 1: Radiomics analysis for choice of optimal location

In order to characterise the local breast textures in the mammographic image such as denser regions and ducts, a radiomics analysis was applied. From all features described in the PyRadiomics documentation^1^, specific features were chosen for their ability to distinguish regions with a certain degree of contrast, coarseness, variance and connected structures. This resulted in the following list of 22 selected features, categorised by type:

- grey level size zone matrix : zone entropy, small area high grey level emphasis ;
- neighbouring grey tone difference matrix : coarseness, contrast, strength ;
- first-order statistics : range, variance, skewness, kurtosis ;
- grey level co-occurrence matrix : cluster prominence, cluster shade, cluster tendency,
- correlation ;
- grey level run length matrix : long run emphasis, grey level variance, long run high
- grey level emphasis, run variance ;
- grey level dependence matrix : large dependence emphasis, dependence non-uniformity,
- dependence variance, dependence entropy, high grey level emphasis.

In the process of finding a plausible location for microcalcification cluster modeling, these features were computed for each grid cell. They were computed with a binwidth equal to 25 and B-spline interpolation. None of the other settings were changed from the default values.

For some specific features the ten grid cells with the lowest values were considered, namely skewness, kurtosis, long run emphasis, run variance and large dependence emphasis. For all other features the ten grid cells with the highest values were considered.

1. van Griethuysen JJM, Fedorov A, Parmar C, et al. Computational radiomics system to decode the radiographic phenotype*. Cancer R*es. 2017;77(21):e104-e107. doi:10.1158/0008-5472.CAN-17-0339

Supplemental 2: Modeling specific clinical types of microcalcification clusters

When opting to simulate a specific clinical type, pre-determined parameters were used. These are based on the properties and descriptions of real calcifications. For both main categories of BI-RADS type for the 3D models four types were implemented with the pre-detemined calcification parameters in table S2.1 and cluster parameters in table S2.2. For the 2D models based on existing breast textures, a distinction between ‘typically benign’ and ‘suspicious morphology’ was made. The pre-set parameters are listed in table S2.3. The large range of circularity of clusters of suspicious morphology denotes any level of circularity is allowed.
