# Supplementary material for: An automated toolbox for microcalcification cluster modeling for mammographic imaging
Source: Med Phys. 2024 Nov 21;52(2):1335–49. doi: 10.1002/mp.17521 (PMC11788264; doi:10.1002/mp.17521)
Supplement: Supplementary file 2 — Supporting Information [file MP-52-1335-s004.doc]

| BI-RADS type | Shape type | Minimum size [mm] | Maximum size [mm] | Minimum noise level | Maximum noise level |
| --- | --- | --- | --- | --- | --- |
| Round | Spherical | (0.30,0.30,0.30) | (0.80,0.80,0.80) | 0.01 | 0.10 |
| Punctate | Spherical | (0.02,0.02,0.02) | (0.50,0.50,0.50) | 0.01 | 0.05 |
| Milk of calcium | Teacup | (0.50,0.50,0.50) | (1.00,1.00,1.00) | 0.01 | 0.10 |
| Large rod-like | Cylindrical | (0.20,0.20,2.00) | (1.00,1.00,6.00) | 0.05 | 0.15 |
| Amorphous | Ellipsoidal | (0.05,0.05,0.05) | (0.25,0.25,0.25) | 0.05 | 0.10 |
| Coarse heterogeneous | Random | (0.50,0.50,0.50) | (0.80,0.80,0.80) | 0.20 | 0.20 |
| Fine pleomorphic | Random | (0.20,0.20,0.20) | (0.50,0.50,0.50) | 0.20 | 0.20 |
| Fine linear | Cylindrical | (0.02,0.02,0.50) | (0.50,0.50,1.00) | 0.05 | 0.20 |

Table S2.1: Parameters for 3D calcification models based on BI-RADS type
