# Supplementary material for: An automated toolbox for microcalcification cluster modeling for mammographic imaging
Source: Med Phys. 2024 Nov 21;52(2):1335–49. doi: 10.1002/mp.17521 (PMC11788264; doi:10.1002/mp.17521)
Supplement: Supplementary file 3 — Supporting Information [file MP-52-1335-s003.doc]

| BI-RADS type | Minimum size [mm] | Maximum size [mm] | Number of micro-calcifications | Minimum distance [mm] | Minimum rotation angles [°] | Maximum rotation angles [°] |
| --- | --- | --- | --- | --- | --- | --- |
| Round | (10,10,10) | (30,30,30) | (5,15) | (0.50,0.50,0.50) | (0,0,0) | (360,360,360) |
| Punctate | (10,10,10) | (30,30,30) | (10,20) | (0.10,0.10,0.10) | (0,0,0) | (360,360,360) |
| Milk of calcium | (10,10,10) | (30,30,30) | (5,15) | (0.50,0.50,0.50) | (0,0,0) | ( 30, 30, 30) |
| Large rod-like | (10,10,20) | (20,20,30) | (5,20) | (0.10,0.10,0.10) | (0,0,0) | ( 30, 30, 30) |
| Amorphous | (10,10,10) | (50,50,50) | (30,50) | (0.10,0.10,0.10) | (0,0,0) | (360,360,360) |
| Coarse heterogeneous | (10,10,10) | (30,30,30) | (15,30) | (0.25,0.25,0.25) | (0,0,0) | (360,360,360) |
| Fine pleomorphic | (10,10,10) | (50,50,50) | (20,50) | (0.10,0.10,0.10) | (0,0,0) | (360,360,360) |
| Fine linear | (10,10,20) | (20, 20,30) | (15,30) | (0.10,0.10,0.01) | (0,0,0) | ( 30, 30, 30) |

Table S2.2: Parameters for 3D microcalcification cluster models based on BI-RADS type
