# Supplementary material for: An automated toolbox for microcalcification cluster modeling for mammographic imaging
Source: Med Phys. 2024 Nov 21;52(2):1335–49. doi: 10.1002/mp.17521 (PMC11788264; doi:10.1002/mp.17521)
Supplement: Supplementary file 4 — Supporting Information [file MP-52-1335-s001.doc]

| BI-RADS type | Minimum calcification size [mm] | Maximum calcification size [mm] | Number of micro-calcifications | Minimum circularity | Maximum circularity |
| --- | --- | --- | --- | --- | --- |
| Typically benign | 0.3 | 1.2 | (10,20) | 0.5 | 1.0 |
| Suspicious morphology | 0.1 | 0.5 | (20,40) | 0.0 | 10.0 |

*Table S2.3: Parameters for 2D microcalcification cluster models based on the clinical type*
